# Distinguishing retinal angiomatous proliferation from polypoidal choroidal vasculopathy with a deep neural network based on optical coherence tomography

**DOI:** 10.1038/s41598-021-88543-7

**Published:** 2021-04-29

**Authors:** Daniel Duck-Jin Hwang, Seong Choi, Junseo Ko, Jeewoo Yoon, Ji In Park, Joon Seo Hwang, Jeong Mo Han, Hak Jun Lee, Joonhong Sohn, Kyu Hyung Park, Jinyoung Han

**Affiliations:** 1 Department of Ophthalmology, Hangil Eye Hospital, 35 Bupyeong-daero, Bupyeong-gu, Incheon, 21388 South Korea; 2 Department of Ophthalmology, Catholic Kwandong University College of Medicine, Incheon, South Korea; 3 Department of Applied Artificial Intelligence, Sungkyunkwan University, 25-2, Sungkyunkwan-ro, Jongno-gu, Seoul, 03063 South Korea; 4 RAON DATA, Seoul, South Korea; 5 Department of Medicine, Kangwon National University Hospital, Kangwon National University School of Medicine, Chuncheon, Gangwon-do South Korea; 6 Seoul Plus Eye Clinic, Seoul, South Korea; 7 Kong Eye Center, Seoul, South Korea; 8 Department of Ophthalmology, Seoul National University Bundang Hospital, Seongnam, South Korea

**Keywords:** Retinal diseases, Medical imaging, Machine learning

## Abstract

This cross-sectional study aimed to build a deep learning model for detecting neovascular age-related macular degeneration (AMD) and to distinguish retinal angiomatous proliferation (RAP) from polypoidal choroidal vasculopathy (PCV) using a convolutional neural network (CNN). Patients from a single tertiary center were enrolled from January 2014 to January 2020. Spectral-domain optical coherence tomography (SD-OCT) images of patients with RAP or PCV and a control group were analyzed with a deep CNN. Sensitivity, specificity, accuracy, and area under the receiver operating characteristic curve (AUROC) were used to evaluate the model’s ability to distinguish RAP from PCV. The performances of the new model, the VGG-16, Resnet-50, Inception, and eight ophthalmologists were compared. A total of 3951 SD-OCT images from 314 participants (229 AMD, 85 normal controls) were analyzed. In distinguishing the PCV and RAP cases, the proposed model showed an accuracy, sensitivity, and specificity of 89.1%, 89.4%, and 88.8%, respectively, with an AUROC of 95.3% (95% CI 0.727–0.852). The proposed model showed better diagnostic performance than VGG-16, Resnet-50, and Inception-V3 and comparable performance with the eight ophthalmologists. The novel model performed well when distinguishing between PCV and RAP. Thus, automated deep learning systems may support ophthalmologists in distinguishing RAP from PCV.

## Introduction

Retinal angiomatous proliferation (RAP) is a subtype of neovascular age-related macular degeneration (AMD), which is one of the leading causes of blindness in elderly people in developed countries^1^. RAP, characterized by retinal-retinal or retinal-choroidal anastomoses^2,3^, has a poor functional prognosis because it is likely to (i) have a poor response to treatment^4,5^ and (ii) develop geographic atrophy, in comparison to other subtypes of neovascular AMD^4,6^. Polypoidal choroidal vasculopathy (PCV) is another subtype of neovascular AMD that manifests as type 1 neovascularization. Indocyanine green angiography (ICGA) shows focal ICGA hyperfluorescence with aneurysmal dilations, also known as polyps^7^. The natural clinical course of PCV is more stable, and its visual outcomes are also known to be more favorable because the incidence of subretinal fibrosis is lower than that of other neovascular AMD types^8,9^. The course of the disease and the response to treatment of PCV was shown to be different from typical AMD forms, and a better response to photodynamic therapy was reported^10^, although the long-term outcome of PCV treatment is still controversial due to the variant of PCV^9–11^. RAP and PCV, as subtypes of neovascular AMD, show similarities and differences^2,12,13^, and an accurate diagnosis is essential to establish an appropriate treatment strategy and to accurately predict a patient’s prognosis^13,14^.

Multimodal imaging has proven to be effective for accurately diagnosing RAP and PCV. Traditionally, the classification of neovascular AMD has been based on fluorescein angiography (FA) or ICGA imaging, which are invasive techniques^2,15^. Optical coherence tomography (OCT) imaging, which is noninvasive and, hence, does not require direct contact, has been used as an adjunct to these angiography methods^13^. Thus, diagnosing RAP and PCV with deep learning techniques using only noninvasive OCT is promising. However, these two disorders are notably difficult to discriminate due to similar OCT findings such as retinal pigment epithelial detachment (RPED)^13^. Although there has been much effort in assessing neovascular AMD using OCT, to the best of our knowledge, no study has reported the use of deep learning techniques to distinguish RAP from PCV.

Recent advances in deep learning techniques, such as convolutional neural networks (CNNs), have provided alternative methods to characterize medical image data. In ophthalmology, previous studies have reported high accuracies in detecting retinal diseases such as central serous chorioretinopathy with CNN-based models using OCT images^16–22^. In the present study, we propose a deep CNN model that uses OCT scans to distinguish RAP from PCV. Additionally, using gradient-weighted class activation mapping (Grad-CAM)^23^ heat maps, specific features determined by the proposed model are visualized to facilitate the understanding of structural differences between RAP and PCV.

## Results

A total of 3951 images from 279 participants were included in the study. The mean age of the participants in the normal group was 64.66 ± 8.42 years, and that of patients in the AMD group was 75.40 ± 8.74 years. Detailed information on the data used in this study is shown in Table 1.

**Table 1:** Baseline characteristics of patients who had undergone macular OCT.

	Normal	Neovascular AMD		
		RAP	PCV	Total
Image, no	2125	862	964	1826
Patients, no	85	107	122	229
Age, years (SD)	64.66 (8.41)	80.35 (6.21)	71.07 (8.33)	75.40 (8.74)
**Gender, no (%)**				
Male	23 (27.06)	20 (18.69)	82 (67.21)	102 (44.54)
Female	62 (72.94)	87 (81.31)	40 (32.79)	127 (55.46)
**Eye, no. (%)**				
Right	44 (51.76)	55 (51.40)	64 (52.46)	119 (51.97)
Left	41 (48.24)	52 (48.60)	58 (47.54)	110 (48.03)

OCT, optical coherence tomography; neovascular AMD, neovascular age-related macular degeneration; RAP, retinal angiomatous proliferation; PCV, polypoidal choroidal vasculopathy; SD, standard deviation.

### Model performance

When distinguishing between AMD and normal cases, the proposed model had 99.1% accuracy, which is higher than that of most of the other well-known CNN models; VGG-16, Resnet, and Inception showed 98.4%, 95.1%, and 99.1% accuracy, respectively. The sensitivity and specificity of the proposed model were 99.2% and 99.1%, respectively (Table 2). In all three cases of incorrectly reading AMD as normal, shallow subretinal fluid was observed, and the model determined these images as normal. In one of these three cases, even though a small CNVM was observed, the model did not recognize it. The four cases where the model misread the normal findings as AMD were all images showing subtle irregularities of the RPE. The area under the receiver operating characteristic curve (AUROC) of the proposed model was 99.9% (95% confidence interval [CI] 0.781–0.960).

**Table 2:** Sensitivity, specificity, and accuracy of the model for detecting neovascular AMD and classifying RAP and PCV OCT images.

	Normal vs. neovascular AMD^a^	
	Predicted normal	Predicted wet AMD
Actual normal	421	4
Actual neovascular AMD^a^	3	365

	RAP vs. PCV	
	Predicted PCV	Predicted RAP
Actual PCV	183	23
Actual RAP^b^	20	169

Neovascular AMD, neovascular age-related macular degeneration; RAP, retinal angiomatous proliferation; PCV, polypoidal choroidal vasculopathy; OCT, optical coherence tomography.

^a^The sensitivity of the classifier for detecting wet AMD eyes was 99.2%, the specificity was 99.1%, and the accuracy was 99.1%.

^b^The sensitivity of the classifier for detecting RAP was 89.4%, the specificity was 88.8%, and the accuracy was 89.1%.

**Figure 1:**
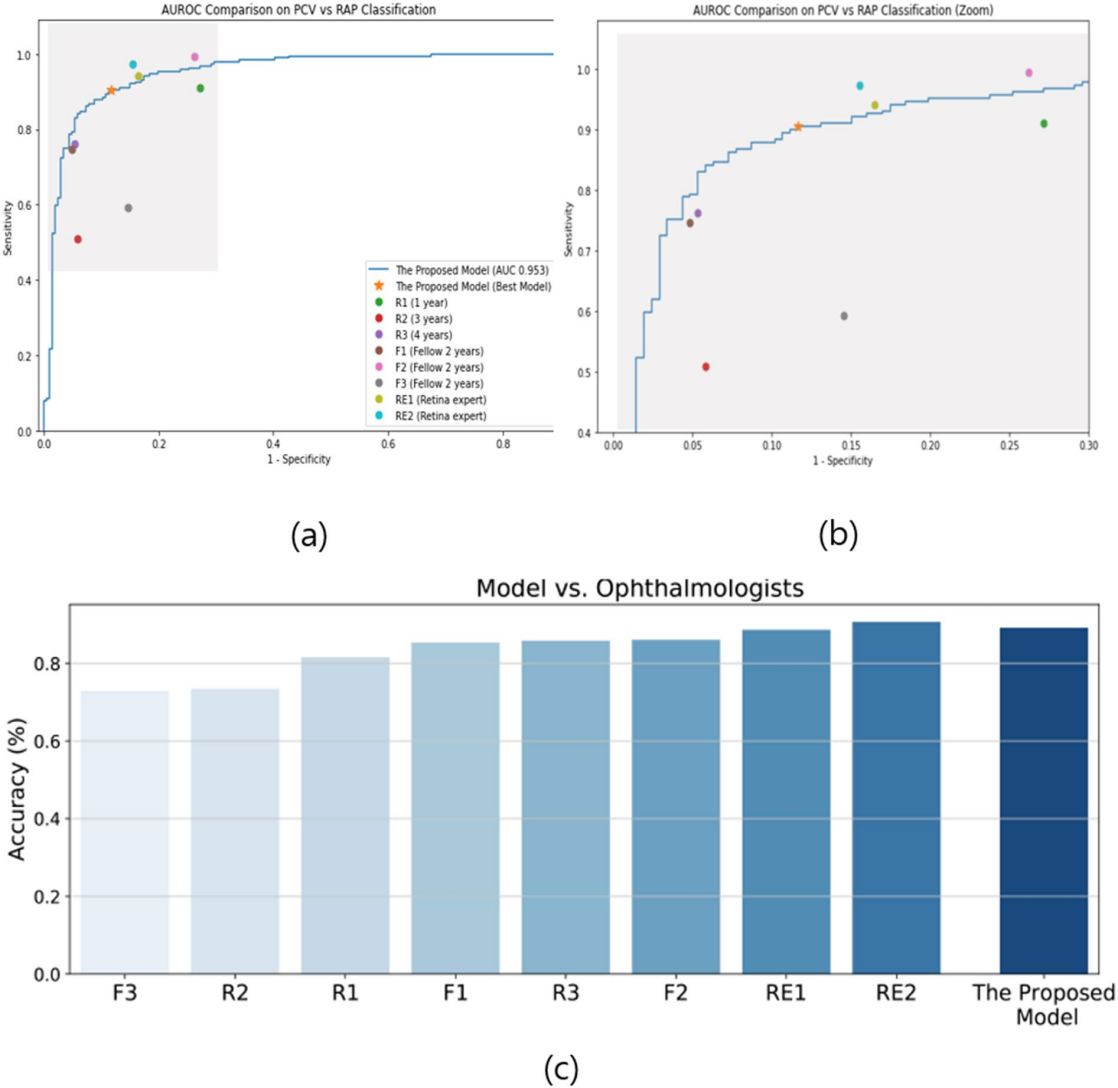
Performances of the classification PCV vs. RAP. (**a**) Performance comparison between our model and the ophthalmologists. The blue line (ROC curve) is created by sweeping a threshold over the predicted probability for a specific clinical diagnosis. (**b**) An expanded version of (**a**). (**c**) Accuracy of our model in comparison to that of the eight ophthalmologists. The performance of our model is better than that of most of these ophthalmologists. AUROC, area under the receiver operating characteristic curve; PCV, polypoidal choroidal vasculopathy; RAP, retinal angiomatous proliferation.

Furthermore, the proposed model had 89.1% accuracy in distinguishing between RAP and PCV, which is again higher than the values of other CNN models; the VGG-16, Resnet, and Inception models showed 87.8%, 66.0%, and 88.1% accuracy, respectively (Fig. 1). The sensitivity and specificity of the proposed model for RAP detection were 89.4% and 88.8%, respectively (Table 2), and its AUROC was 95.3% (95% CI 0.727–0.852). Table 3 shows six representative images of cases that our model classified correctly even though they were misclassified by the two retina experts.

**Table 3:** Examples of decisions made by the model and 8 human experts.

						
Model	PCV	PCV	PCV	PCV	PCV	RAP
R1	RAP	RAP	PCV	PCV	PCV	PCV
R2	PCV	RAP	PCV	PCV	PCV	PCV
R3	PCV	PCV	PCV	PCV	PCV	PCV
F1	PCV	PCV	PCV	PCV	PCV	PCV
F2	PCV	RAP	PCV	RAP	RAP	RAP
F3	RAP	RAP	RAP	PCV	PCV	PCV
RE1	RAP	RAP	RAP	RAP	RAP	PCV
RE2	RAP	RAP	RAP	RAP	RAP	PCV
GT	PCV	PCV	PCV	PCV	PCV	RAP

These 6 images are the cases, which our model classified correctly even though two retina experts classified incorrectly.

PCV, polypoidal choroidal vasculopathy; RAP, retinal angiomatous proliferation; R1, R3 and R4 denote ophthalmology residents with 1 year, 3 years, and 4 years of experience, respectively. F1, F2, and F3 denote retina fellows with 2 years of experience. RE1 and RE2 refers to retina experts with more than 10 years of experience as retina experts. GT denotes the ground truth.

### Performance comparison with ophthalmologists

**Figure 2:**
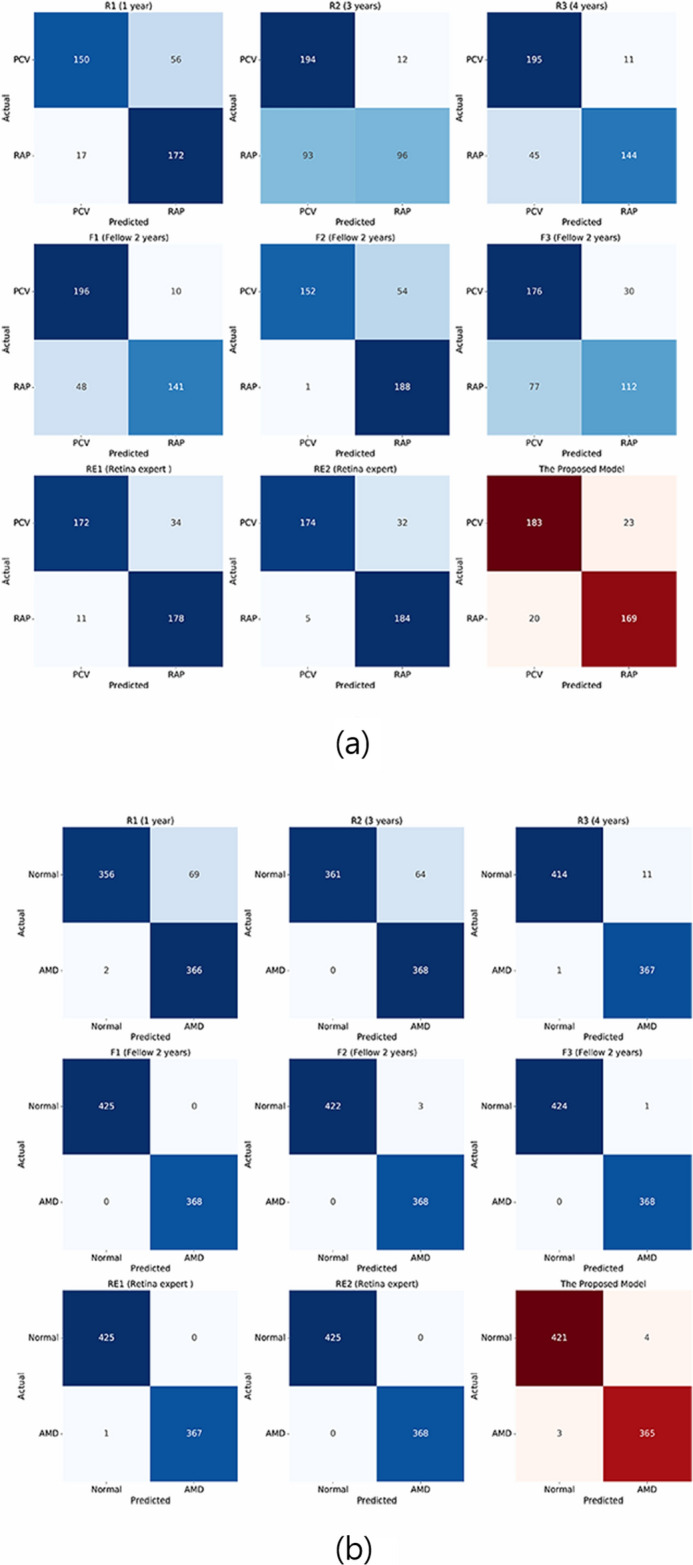
Confusion matrixes of the eight ophthalmologists and the proposed model. (**a**) Normal retina vs. neovascular AMD. Confusion matrixes showing how the eight ophthalmologists and the model classified the test set. Five of the eight ophthalmologists have an accuracy of more than 99%, whereas the model shows 99.1% accuracy. (**b**) Confusion matrixes of distinguishing between PCV and RAP cases. The average accuracy of the eight ophthalmologists is 83.0%, and our proposed model has an accuracy score of 89.1%. R1, R2, and R3 denote ophthalmology residents with 1 year, 3 years, and 4 years of experience, respectively. F1, F2, and F3 denote retina fellows with 2 years of experience. RE1 and RE2 refer to retina experts with more than 10 years of experience at a retina clinic.

To compare ophthalmologists’ and our proposed model’s performance in the classification of AMD and normal cases, the same 793 images were provided to eight ophthalmologists and our proposed model. Similarly, to compare their performance in distinguishing PCV from RAP cases, the same 395 images were used. In the categorization of AMD and normal cases, the classification accuracies of the ophthalmologists ranged between 92.8 and 100%. The average accuracy of the eight ophthalmologists was 97.6%, whereas that of the proposed deep learning model was 99.1%. In the discrimination between RAP cases and PCV cases, the classification accuracies of the ophthalmologists ranged from 72.9 to 90.1%. The average accuracy of the ophthalmologists was 83.0%, whereas the proposed deep learning model showed 85.2% accuracy. The performance comparison between the proposed model and the eight ophthalmologists (three residents, three fellows, and two retina experts) is shown in Fig. 1. Among the eight ophthalmologists, the kappa coefficient for the two retina experts with more than 10 years of experience was 0.867 in distinguishing between RAP and PCV cases. This indicates that the two retina specialists discriminated RAP from PCV cases using similar criteria. The kappa coefficients between the proposed model and each of the two retina specialists were 0.81 and 0.78, respectively. This suggests that the proposed model made judgments similar to those of the retina experts when distinguishing between RAP and PCV cases. We identified 35 cases that were correctly categorized by the two retina experts but incorrectly classified by three or more of the remaining six ophthalmologists, implying that those cases required professional experience in retinal disorders. Out of these 35 cases, the proposed model correctly classified 31 cases, signifying that the model could play a subsidiary role in the distinction between PCV and RAP. Figure 2 shows how the eight ophthalmologists and the suggested model classified the test set. The x-axis denotes the predicted class (by model), and the y-axis denotes the actual class of a given OCT image. As shown in Fig. 2, our proposed model had a 99.1% accuracy score. In distinguishing between PCV and RAP cases, the model showed 89.1% accuracy.

### Evaluation of the RAP classification process using Grad-CAM

**Figure 3:**
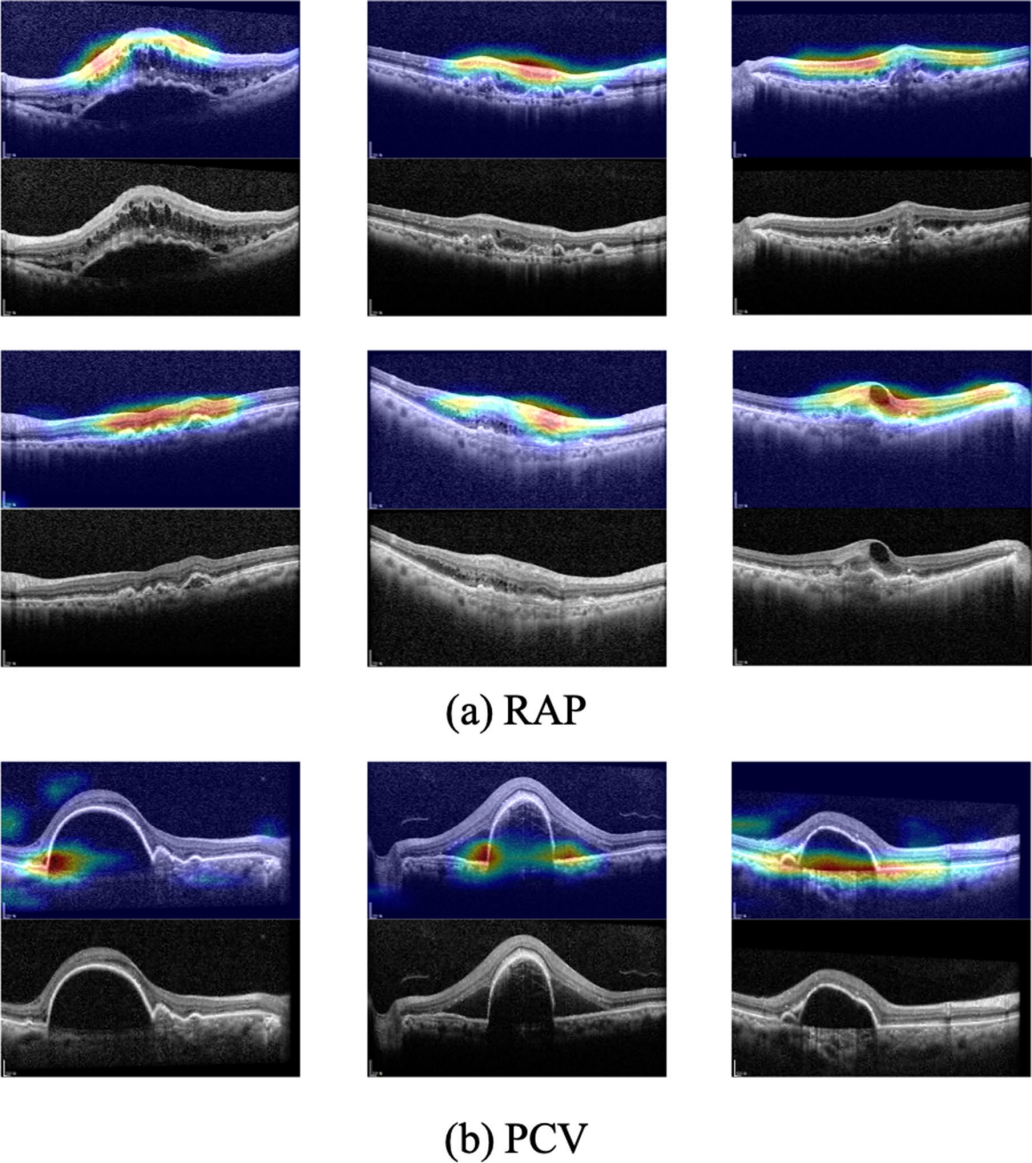
Grad-CAM of the model for six RAP images and three PCV images. The Grad-CAM images display the heat maps of regions used by the model for classifying PCV and RAP images. Grad-CAM was able to identify pathologic regions on the spectral-domain optical coherence tomography images. In PCV cases, Grad-CAM emphasized the sharp edge of the steeper RPED. In one of these PCV cases, the Bruch membrane directly under the RPED was also emphasized. Grad-CAM, gradient-weighted class activation mapping; RAP, retinal angiomatous proliferation; PCV, polypoidal choroidal vasculopathy; RPED, retinal pigment epithelial detachment.

The representative heat maps generated by Grad-CAM in the RAP classification process are shown in Fig. 3. The marked areas in the heat maps are regions in which the model recognized important features. These highlighted regions were similar to the regions that retina specialists usually examine when diagnosing RAP cases. In other words, the proposed model used a similar approach in the assessment of RAP cases.

## Discussion

Our study was the first attempt to differentiate between RAP and PCV using a CNN model without segmentation. The proposed model showed results comparable to those of ophthalmologists with various levels of experience, i.e., ophthalmology residents, retina fellows, and retina experts. The model was able to distinguish between normal findings and AMD (PCV or RAP) with a very high accuracy of 99.1%, and as in previous reports^17–19^, it was shown that deep learning models can successfully be used for screening purposes to differentiate between normal findings and AMD. Regarding the classification of RAP and PCV, the accuracy was 89.1%. This was comparatively lower than the model accuracy for differentiating between normal retinas and AMD, but the accuracy of the model was not inferior to that of the ophthalmologists. The model results were even comparable to those of the two retina experts.

RAP is known to have a poor prognosis due to the relatively high risk of bilateral involvement and poor response to anti-VEGF injection therapy^4–6,24^. Therefore, it is important to accurately diagnose RAP, distinguish it from other neovascular AMDs, and determine the specific prognosis before starting treatment. By contrast, PCV is known to have a relatively better prognosis than other neovascular AMDs, although no study has directly compared PCV with RAP^8,25–27^. Traditionally, RAP and PCV have been diagnosed based on FA and ICGA findings, and OCT has been used as an auxiliary source of information. Recently, attempts have been made to diagnose RAP and PCV only using OCT by identifying image characteristics specific for RAP^13^ or PCV^9^. In OCT images, RAP and PCV show similar patterns, such as RPED, making it difficult even for an experienced ophthalmologist to distinguish between PCV and RAP based only on OCT findings. RAP has distinct OCT features such as intraretinal cyst-like fluid accumulation^28–30^, a break of the RPED^28,30^, gently-sloping dome-shaped or trapezoid-shaped RPEDs without obvious peaks^13^, intraretinal or intraretinal-subretinal hyperreflective mass lesions^28,31^, and a thin choroid^32,33^. In PCV, findings such as sharp-peaked RPEDs^34,35^, sub-RPE ring-like lesions^9,35^, complex or multilobular PEDs^9,34^, and notched RPEDs^34,35^ are known as revealing OCT patterns. Because FA and ICGA are time-consuming and invasive tests, attempts to distinguish between PCV and RAP using only OCT are considered promising. It may be premature to assume that OCT can completely replace FA and ICGA. But it would be beneficial if deep learning models could differentiate between the two diseases using only OCT images because FA or ICGA would not have to be performed in every affected patient. Besides, if a deep learning model had a good performance in distinguishing between the two diseases, this would be an important step toward developing a model that can identify several subtypes of neovascular AMD using only OCT images.

Currently, it remains difficult to distinguish between RAP and PCV using only a single OCT image. This is supported by our results of the OCT image readings by the ophthalmologists. Although the two retina experts showed the best results among the eight ophthalmologists, the results of the residents and retina fellows did not reveal substantial differences, demonstrating the difficulties to discriminate between RAP and PCV if only one OCT image is available. As shown in the confusion matrixes of RAP vs. PCV in Fig. 2b, some ophthalmologists (R2, R3, F1, F3) mainly judged RAP cases incorrectly as PCV cases. By contrast, other ophthalmologists (R1, F2) mainly misdiagnosed PCV as RAP. This indicates that ophthalmologists might have their own criteria for OCT findings to distinguish the two diseases, and the inaccuracy might be increased partially due to the limitation of basing a judgment on only one OCT image.

Recently, Kim et al.^13^ described the RAP characteristics of OCT images. RPED is commonly observed in both RAP and PCV, but the morphological RPED characteristics differ between RAP and PCV. According to their report, gently-sloping dome-shaped or trapezoid-shaped RPEDs without an obvious peak are apparent in RAP. The authors demonstrated these characteristic RPED features using representative images that were selected from several OCT image cuts with RAP-specific findings. Among the cuts of patients’ lesions in our study were critical and significant cuts that showed characteristic RAP or PCV findings similar to those reported by Kim et al.^13^. Since our test data also included other, relatively noncritical cuts, it was more difficult for both ophthalmologists and the deep learning model to distinguish between the two diseases based on only one noncritical OCT cut. Nevertheless, the proposed deep learning model showed a performance comparable to that of the ophthalmologists. In interpreting these results, it is worth noting that the proposed deep learning model showed a performance similar to that of ophthalmologists in distinguishing the two diseases based on only one OCT image. If multiple OCT images were provided, the classification accuracy of the ophthalmologists would certainly increase, but in the case of the deep learning model, a further investigation on its use of multiple OCT images for the diagnostic process would be necessary in future studies.

If the deep learning model accurately distinguished RAP from PCV based on only one OCT image, the model could support ophthalmologists in their interpretation of OCT images. Among the images that most ophthalmologists misclassified as RAP or PCV, 35 cases were correctly determined by the two retina experts, whereas the proposed model classified 31 (89%) of those cases correctly. Moreover, the kappa coefficients were high between the two retina experts, as well as between the deep learning model and each of the retina experts. This suggests that the proposed deep learning model can support ophthalmologists who are not retina experts in their diagnosis of RAP and PCV, a task that requires the involvement of skilled retina experts. Although 28 cases were wrongly diagnosed by both retina experts, the model was correct in 11 (39%) of these cases, suggesting that the proposed deep learning model can also assist retina experts in their judgment.

In certain macular diseases such as AMD, we suggest Grad-CAM^23^ as an adjunctive tool for the detection of distinct macula features as OCT biomarkers^22^. The Grad-CAM results generated by the deep learning model are in agreement with the findings by Kim et al.^13^ regarding the presence of (i) intraretinal fluid accumulation, (ii) gently-sloping dome-shaped RPEDs without an obvious peak, and (iii) intraretinal mass lesions, which all can be considered to be distinct features that can distinguish RAP from PCV. This reveals that the model developed criteria similar to those used by ophthalmologists; it can be presumed that the proposed model learned to detect important OCT biomarkers that can distinguish between the two diseases. Interestingly, the thinning of the choroid was largely not considered by the proposed model for the classification of the two diseases; in contrast to the retina, the choroid was rarely highlighted on the heat map. Several studies^13,32,33^ reported that thinning of the choroid is a characteristic feature of RAP. In the study by Kim et al.^13^, choroidal thinning of < 200 µm was not observed in about 20% of all RAP cases, although the criterion of a thinned choroid is not well defined in clinical practice. The Grad-CAM findings of our study suggest that the choroid was not an essential feature used by the proposed model to distinguish between RAP and PCV. Future studies should investigate whether choroidal findings have implications for the development of a deep learning model that can differentiate between PCV and RAP.

To train our deep learning model with a limited number of spectral-domain (SD)-OCT images, we applied transfer learning^36^. Transfer learning is a widely used technique in which a model starts training from pre-trained weights (from large-scale data) and updates its weights on the target task, i.e., to distinguish between OCT images of RAP and PCV cases. It should be noted that we used ImageNet data as our pre-trained set because it contains large-scale and high-quality images. As a result, the proposed model with transfer learning had higher performance scores than the model without transfer learning. This implies that transfer learning can play an important role in assessing OCT images if only a small number of images are provided.

In image-based classification tasks (e.g., PCV vs. RAP) by deep learning models, data augmentation is commonly performed based on simple parameterized transformations, such as image rotation and scaling, which can reduce overfitting and improve the model performance in various areas^37–39^. More specifically, image data augmentation is a technique that generates new images by flipping or rotating original images. The rationale behind medical image augmentation is that the augmented (i.e., newly generated) images maintain the disease-related information of the original image but transform this information into different shapes. To build a robust model for classifying RAP and PCV, we applied the following data augmentation. First, we flipped the training set images horizontally. Second, we moved the images horizontally and vertically. Third, we tilted the original images. As a result, the performance of the proposed model using the augmented data has been significantly improved, which confirms that the data preprocessing methods used in our study were useful in distinguishing between RAP and PCV with a relatively small dataset.

Our study has several limitations. First, the number and variety of available OCT images were limited. External validation needs to be performed in future studies because all images in this study were acquired from a single hospital. However, the dataset was sufficient to demonstrate the feasibility of the proposed deep learning model to distinguish RAP from PCV using OCT images. Second, the number of the patients with PCV was much higher than that of patients with RAP in our hospital’s cohort dataset. To address such an imbalance, we applied down-sampling, that is a widely-used technique, to allow robust learning for our model and avoid performance degradation. Therefore, external validation in future studies is warranted. Third, the proposed model performed the binary classification for two diseases, i.e., RAP vs. PCV. While we focused in this study on classifying two diseases, we suggest that it can be the basis for the development of a deep learning model that can classify multiple types of AMD. This question should be addressed in future studies. Fourth, we examined the performance of the model using only one, not multiple OCT images. In clinical practice, ophthalmologists usually make a comprehensive diagnosis by looking at several OCT images of the same patient. For the diagnosis of RAP or PCV, it would be better to combine multiple images than to base the judgment on only one isolated OCT image. Hence, we plan to develop a deep learning model that utilizes multiple OCT images or an OCT image with additional multimodal images (e.g., FA, ICGA, infrared reflectance). This may improve the performance in comparison to the current model. Lastly, this was a cross-sectional study. The model could be extended toward predicting treatment response or lesion progression based on a series of OCT images. In addition to determining the current status by analyzing the latest image, the extended model could predict future disease progression of patients with RAP or PCV using their longitudinal image data. Lastly, since we did not find any similar investigations or prior work using only OCT images for the classification of RAP and PCV, we could not compare the performance of our model with that of previously published models. Despite these limitations, the proposed model demonstrated a promising diagnostic value suggesting the need for further investigations on its potential impact on the clinical diagnosis of RAP and PCV.

In summary, we developed a deep learning CNN model that performed well in distinguishing between RAP and PCV using only OCT images. Automation of the classification process with this deep learning model may support both retinal experts and non-experts in their task to distinguish between PCV and RAP. We believe that this study formed the basis for further investigations to develop accurate OCT-based deep learning models with high performance for the diagnosis of RAP and PCV.

## Methods

### Ethics statement

This study was conducted in accordance with the Helsinki Declaration of 1964. The Ethics Committee of Hangil Eye Hospital approved the research protocols and their implementation (IRB #: Hangil IRB-20007). The committee waived the requirement for informed consent given that this was a retrospective observational study of medical records and that it was retrospectively registered.

### Data collection and labeling

We analyzed the records of patients who visited Hangil Eye Hospital between January 2014 and January 2020. We used SD-OCT (Heidelberg Spectralis; Heidelberg Engineering, Heidelberg, Germany) images of normal healthy participants and patients with either RAP or PCV. Among the 229 patients enrolled at the outpatient clinic during that period, 107 were newly diagnosed with RAP, and 122 were newly diagnosed with PCV. Additionally, 85 subjects were assigned to the normal healthy group. All RAP and PCV cases were diagnosed by independent retina specialists using fundus examinations, FA, ICGA, and OCT images. A confocal scanning laser ophthalmoscope (Heidelberg Retina Angiograph HRA; Heidelberg Engineering, Heidelberg, Germany) was used to simultaneously perform FA and ICGA in all patients with RAP or PCV. One eye per patient was selected for this study, with one visit per patient. We used only OCT images of treatment-naïve eyes before intravitreal anti-vascular endothelial growth factor (VEGF) injection.

**Figure 4:**
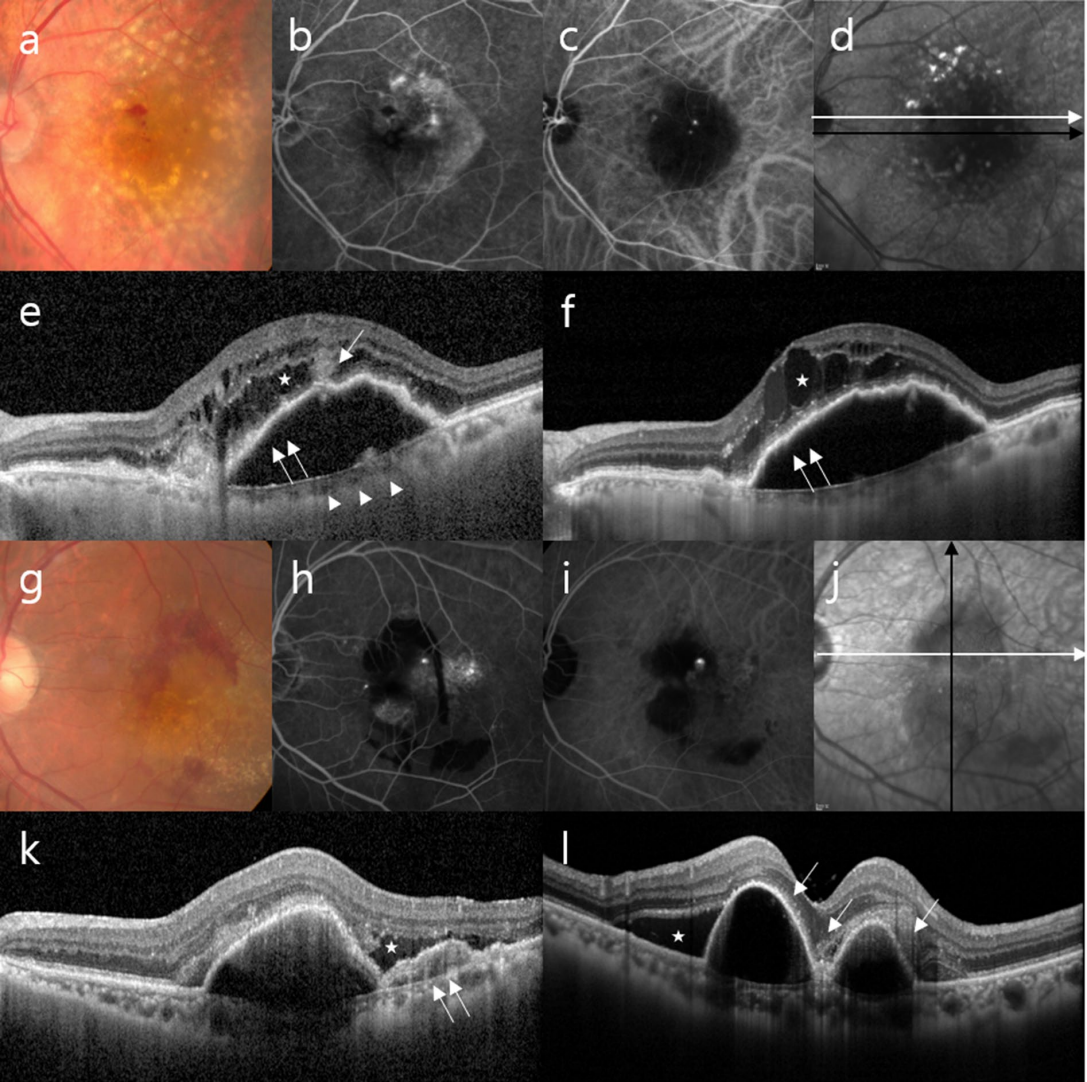
Representative cases of RAP and PCV. (**a**–**f**) RAP. (**a**) Fundus photography, (**b**) FA, (**c**) ICGA, (**d**) infrared reflectance, and (**e**,**f**) OCT images of an eye with RAP. The OCT images (**e**,**f**) show typical features of RAP: a thin choroid (arrowheads), intraretinal cyst-like fluid accumulation (asterisks), absence of subretinal fluid, trapezoid-shaped RPED (double arrows), and the presence of an intraretinal-subretinal continuous hyperreflective lesion with a break in the RPE (arrow). The white and black arrows in the infrared reflectance image (**d**) indicate the OCT scanning lines for the images (**e**) and (**f**), respectively. (**g**–**l**) PCV. (**g**) Fundus photography, (**h**) FA, (**i**) ICGA, (**j**) infrared reflectance, and (**k**,**l**) OCT images of an eye with PCV. The OCT images (**k**,**l**) show irregular retinal pigment epithelium elevation with double-layer sign (double arrow), subretinal fluid (asterisks), and sharp-peaked or steeper dome-shaped RPEDs or notches (arrows). The white and black arrows in the infrared image (**j**) indicate the OCT scanning lines for the images (**k**) and (**l**), respectively. FA, fluorescein angiography; ICGA, indocyanine green angiography; OCT, optical coherence tomography; PCV, polypoidal choroidal vasculopathy; RAP, retinal angiomatous proliferation; RPE, retinal pigment epithelium; RPED, retinal pigment epithelial detachment.

The FA-/ICGA-based classification of neovascular AMD was performed by two retina specialists (DDH and HJL) who reviewed the medical charts and examined all images obtained by OCT, FA, and ICGA multimodal imaging. In cases of disagreement, a third retina specialist (JS) assessed the discrepancy and discussed the case with other specialists. All discrepancies were resolved by consensus. Cases that exhibited retinal-retinal or retinal-choroidal anastomoses were classified as type 3 neovascularization, i.e., RAP (Fig. 4a–f). PCV was diagnosed based on the presence of branching vascular networks and/or terminating polypoidal lesions (Fig. 4g–l). Other cases were classified as typical neovascular AMD with type 1 or type 2 choroidal neovascularization and excluded from this study. Our analysis also excluded data that showed the presence of other potentially conflicting retinal pathologies such as central serous chorioretinopathy, diabetic retinopathy, and branch retinal vein occlusion.

Central volume scans using a 25-scan pattern and macular thickness map protocols were routinely performed subsequently using the SD-OCT scanner of our hospital. Through this process, a volumetric assessment of the central retinal structures consisting of 25 single horizontal axial scans was routinely done (scanning area: 6 × 6 mm, centered at the fovea), as well as horizontal or vertical scans. Although horizontal or vertical scan images were available, we did not use them in this study. Instead, we only used the central volume scans comprised of 25 images. To extract the lesion cuts that were used for training and testing, all lesion cuts from the 25 SD-OCT images were first selected for each patient by a retinal specialist (DDH). Thereafter, *N* (i.e., $0\le N\le 5$) lesion cuts located between the 11th and 15th cut (i.e., central region) were selected, as well as $10-N$ non-centered lesion cuts (including the parafoveal or perifoveal area) located between either the 1st and 10th cut or the 16th and 25th cut, which were however selected at random instead. When the number of existing non-centered lesion cuts was < $10-N$, all non-centered lesion cuts were selected. Therefore, up to 10 images were selected per patient in this study.

### Data preprocessing

To use the SD-OCT images as input for a deep CNN, we first removed unnecessary parts, e.g., company logo, date, and time, from each SD-OCT image. We cropped the SD-OCT images to RGB images of size 490 × 764 and then downsampled them into 224 × 224 RGB images for the deep CNN, which only takes fixed-size images as inputs. It should be mentioned that the 224 × 224 RGB format is a widely used image shape that is popular in deep learning models for image classification, such as VGG-16^40^, VGG-19^40^, Resnet^41^, and Inception^41^.

To build a robust classification model, data augmentation was applied in the training phase. New images were generated from existing ones so that the proposed model can be trained with a variety of images. In particular, we generated new images by (i) flipping images horizontally, (ii) moving images horizontally and vertically by 10% of the image size, and (iii) rotating the original images up to 15°.

### Model architecture

**Figure 5:**
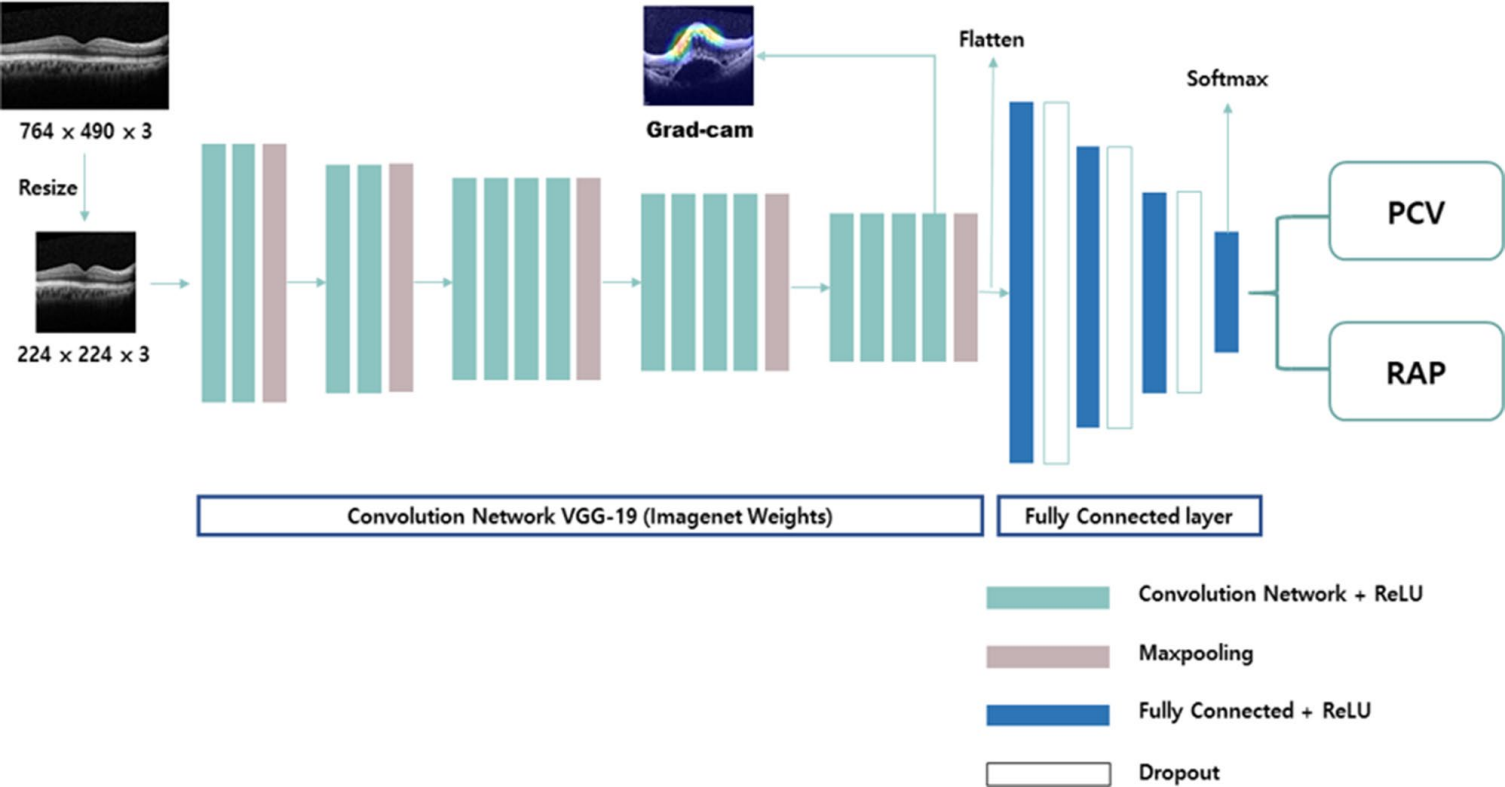
Network architecture of the modified VGG-19 used for classifying PCV and RAP. Our model consisted of an input layer, 16 CNN layers with ReLU activation functions, 5 max-pooling layers, 3 dropout layers, and 4 fully connected layers with ReLU activation functions. The last fully connected layer was used for binary classification. The heat map was generated from the final CNN layer. CNN, convolutional neural network; Grad-CAM, gradient-weighted class activation mapping; PCV, polypoidal choroidal vasculopathy; RAP, retinal angiomatous proliferation; ReLU, rectified linear unit.

To classify a given OCT image as (i) either neovascular AMD or normal and (ii) either PCV or RAP, we built the OCT image classification model based on the VGG-19^40^ architecture. We considered other well-known CNN architectures, including VGG-16^40^, Resnet^40,41^, and Inception^42^, but we decided to use the VGG-19 because it performed better than others. Originally, the VGG-19 architecture contains 19 trainable layers, including convolution, fully connected, max-pooling, and dropout layers. In the model proposed in this study, we replaced the 3 fully connected layers of the original VGG-19 with 4 fully connected layers with 3 dropout layers, as shown in Fig. 5. The proposed model consisted of 16 convolutional network layers with a rectified linear unit (ReLU) activation function, 5 max-pooling layers, and 4 fully connected layers. The final output layer with a soft-max activation function was used to predict the binary classification result, i.e., the PCV or RAP diagnosis. To improve the classification accuracy, we applied the transfer learning approach^36^, which allocated the weights of the ImageNet pre-trained model to the weights of the convolution network in our proposed model. Applying transfer learning proved to be effective as it increased the accuracy of the proposed model by 3%.

### Gradient-weighted class activation mapping

We used Grad-CAM^22^ to visualize potentially pathologic regions of an OCT image. To visualize the important regions of an image for the prediction of the target label, Grad-CAM extracts the gradients of the target label (i.e., RAP and PCV) with respect to feature maps of the convolutional layer, resulting in a heat map that shows the image area on which the model focused during the classification process.

### Experimental setup

We first randomly selected 80% and 20% of the data as the training and test sets, respectively. In classifying AMD and normal cases, the training and test sets were 3158 images (AMD: 1458, normal: 1700) and 793 images (AMD: 368, normal: 425), respectively. In distinguishing between PCV and RAP cases, the training and test sets were 1431 images (PCV: 758, RAP: 673) and 395 images (PCV: 206, RAP: 189), respectively. The test set was only used for the final evaluation of the model performance, and any patient included in the training set was not included in the test set.

To fairly compare the proposed model with the three well-known CNN architectures, i.e., VGG-16, Resnet, and Inception, all models were trained with the same hyperparameters; the batch size and epoch count were 64 and 100, respectively, and the loss function was the categorical cross-entropy with Adam optimization (learning rate: 0.0001). The four models were evaluated using the same test set. Additionally, to assess the proposed model from a clinical perspective, the classification results for the test set were compared with those of eight ophthalmologists, including three ophthalmology residents, three retina fellows, and two retina experts, the latter each with more than 10 years of clinical experience at an academic retina center.

### Statistical analysis

To measure the performance of the model, the sensitivity, specificity, accuracy, and AUROC^43^ were determined. Cohen’s kappa coefficients were used to rate the agreement level between the two retina experts and between the proposed model and each expert.

## Data Availability

The data are not available for public access because of patient privacy concerns but are available from the corresponding authors (DDH and JYH) on reasonable request.
